# Motivational Modulation of Age-Related Effects on Reaching Adaptation

**DOI:** 10.3389/fpsyg.2018.02285

**Published:** 2018-11-20

**Authors:** Jing Huang, Mathias Hegele, Jutta Billino

**Affiliations:** ^1^Abteilung Allgemeine Psychologie, Justus-Liebig-Universität Gießen, Giessen, Germany; ^2^Experimentelle Sensomotorik, Justus-Liebig-Universität Gießen, qGiessen, Germany

**Keywords:** motor learning, visuomotor adaptation, reaching, healthy aging, motivation

## Abstract

Previous studies have provided consistent evidence that adaptation to visuomotor rotations during reaching declines with age. Since it has been recently shown that learning and retention components of motor adaptation are modulated by reward and punishment, we were interested in how motivational feedback affects age-related decline in reaching adaptation. We studied 35 young and 32 older adults in a reaching task which required fast shooting movements toward visual targets with their right hand. A robotic manipulandum (vBOT system) allowed measuring reaching trajectories. Targets and visual feedback on hand position were presented using a setup that prevented direct vision of the hand and projected a virtual image by a semi-silvered mirror. After a baseline block with veridical visual feedback we introduced a 30° counterclockwise visuomotor rotation. After this adaptation block we also measured retention of adaptation without visual feedback and finally readaptation for the previously experienced rotation. In the adaptation block participants were assigned to one of three motivational feedback conditions, i.e., neutral, reward, or punishment. Reward and punishment feedback was based on reaching endpoint error. Our results consistently corroborated reduced motor learning capacities in older adults (*p* < 0.001, η^2^ = 0.56). However, motivational feedback modulated learning rates equivalently in both age groups (*p* = 0.028, η^2^ = 0.14). Rewarding feedback induced faster learning, though punishing feedback had no effect. For retention we determined a significant interaction effect between motivational feedback and age group (*p* = 0.032, η^2^ = 0.13). Previously provided motivational feedback was detrimental for young adults, but not for older adults. We did not observe robust effects of motivational feedback on readaptation (*p* = 0.167, η^2^ = 0.07). Our findings support that motor learning is subject to modulation by motivational feedback. Whereas learning is boosted across both age groups, retention is vulnerable to previously experienced motivational incentives in young adults. In summary, in particular older adults benefit from motivational feedback during reaching adaptation so that age-related differences in visuomotor plasticity, though persisting, can be attenuated. We suggest that the use of motivational information provides a potentially compensatory mechanism during functional aging.

## Introduction

The current understanding of functional aging processes is dominated by a strong focus on cognitive capacities (for reviews, see Hasher and Zacks, 1988; West, 1996; Baltes et al., 1999; Park and Reuter-Lorenz, 2009). Whereas sophisticated models for cognitive decline and the underlying neural mechanisms have been developed, visuomotor changes across the adult lifespan are still not well understood. However, they provide a unique opportunity to investigate the complexity of functional aging in which sensory, motor, cognitive, and also motivational processes interact. Although most cognitive theories share the general assumption of a core primitive of aging that ultimately results in global decline, there is emerging evidence that emphasizes the need to investigate developmental changes considering the complexity of interwoven functional subprocesses that ultimately shape behavioral capacities (van den Bos and Eppinger, 2016). This comprehensive approach might contribute to improving aging models in order to differentiate between decline and stability across the lifespan.

Plasticity of visuomotor behavior represents a crucial capacity in a continuously changing environment and a number of studies have explored age-related changes. Previous studies were in particular concerned with motor adaptation, i.e., the recalibration of well-trained movements to changes in the environment. Motor adaptation thus represents a basic type of motor learning that is generally distinguished from skill acquisition which refers to learning of novel movement patterns (Wolpert et al., 2011; Diedrichsen and Kornysheva, 2015). Note that in the context of motor adaptation learning is frequently inferred from both changes in performance during adaptation as well as subsequent retention and transfer tests. In contrast, skill acquisition, characterized as a persistent change in the behavioral capabilities of the learner, has typically been assessed in retention and transfer tests in order to obtain purer measures of the newly acquired skill independent of practice-related variables (Schmidt and Lee, 2011; Sternad, 2018). This distinction should be kept in mind to avoid confusions. Typical motor adaptation paradigms elicit gradual changes in eye or reaching movements in order to compensate for manipulated visual feedback on movement outcome or disturbing force fields during movement execution. Age effects on motor adaptation have been primarily investigated in reaching paradigms introducing a visuomotor rotation (for review, see Bock and Schneider, 2002).

Congruent with knowledge on cognitive plasticity decline with increasing age (for review, see Jones et al., 2006), numerous studies on reaching adaptation in different age groups have provided overall support for an age-related vulnerability (e.g., Buch et al., 2003; Bock, 2005; Seidler, 2006; Heuer and Hegele, 2008b). However, it has remained ambiguous which processes contribute to the observed deterioration and which processes are robust to decline, thus potentially stabilizing adaptation capacities. Indeed several findings have suggested that reaching adaptation might be subject to only minor decline or can be even preserved across the lifespan depending on specific experimental conditions (Bock and Schneider, 2001; Heuer and Hegele, 2008a; Cressman et al., 2010). These results suggest that adaptation can be driven by a variety of processes that differ in vulnerability during aging. Consequently, vivid efforts have emerged aimed at identifying critical modulators of age-related decline in motor plasticity.

A particularly important distinction has been made between implicit and explicit knowledge fuelling visuomotor adaptation. Implicit knowledge eludes consciousness and builds up gradually across adaptation. In contrast, explicit knowledge of visuomotor perturbations triggers intentional, conscious movement corrections in order to compensate for the experienced error. The availability and efficiency of this adaptation component is closely linked to executive resources and cognitive strategies. Using paradigms that allowed dissociating both components, consistent evidence has been provided that aging is detrimental to explicit adaptation processes, whereas implicit adaptation processes remain remarkably stable across the lifespan (Bock and Girgenrath, 2006; Heuer and Hegele, 2009; Hegele and Heuer, 2010a,b, 2013; Heuer et al., 2011; Huang et al., 2017). Another candidate modulator of age-related effects on motor adaptation has been suggested recently by a study on the role of reinforcement mechanisms during adaptation (Heuer and Hegele, 2014). Findings indicated that a reduced efficiency of motivational mechanisms might add to the decline in adaptive capacities.

This evidence for the impact of motivational factors on age-related changes in motor adaptation appears intriguing because there is a coincidence with accumulating recent work on the effects of reward and punishment during motor adaptation. In the traditional understanding of motor adaptation as a most basic mechanism of motor control, the effects of motivational feedback on plasticity were assumed to be negligible (e.g., Mazzoni and Krakauer, 2006; Wolpert et al., 2011). However, this assumption has been questioned by several findings. A number of studies have supported beneficial effects of motivational feedback, in particular reward, on motor adaptation (Nikooyan and Ahmed, 2015; Kojima and Soetedjo, 2017; Quattrocchi et al., 2017; but see van der Kooij and Overvliet, 2016). In addition, Galea et al. (2015) disentangled differential contributions of reward and punishment to learning and retention during adaptation. Their results showed that punishment was associated with faster learning, but reward boosted retention when visual feedback on movement outcomes was withdrawn. In summary, motivational feedback qualifies for a substantial modulator of motor adaptation. The question arises how this functional link is affected by increasing age.

The impact of motivational processing on behavioral control during aging has been intensively investigated over the last years, however, almost exclusively in cognitive learning or decision paradigms. Findings suggested a reduced sensitivity to reward and punishment with increasing age, resulting in an attenuation of motivational feedback effects on behavior (e.g., Brown and Ridderinkhof, 2009; Eppinger et al., 2011, 2013; Mata et al., 2011; Bauer et al., 2013). Heterogeneity of results, though, points to a strong dependence on task characteristics and the critical role of cognitive control processes. It remains to be clarified whether age-related changes in motivational mechanisms also challenge motor control. Neural correlates of motivational processing in particular include dopaminergic neuromodulation and connectivity between brain networks (Montague et al., 2004; Ernst and Fudge, 2009). These are known to be subject to pronounced age-related decline (Bäckman et al., 2010; Sala-Llonch et al., 2015; Samanez-Larkin and Knutson, 2015). Thus, the effects of motivational manipulations on motor performance might differ between age groups.

We aimed to investigate how motivational incentives modulate age-related differences in motor learning. Using an established visuomotor rotation paradigm that is known to robustly induce adaption in reaching movements, we compared motor plasticity between young adults and healthy, community-dwelling older adults. In addition, within each age group we provided neutral, rewarding, or punishing feedback when adapting for reaching endpoint errors. We expected to observe age-related decline in reaching adaptation, but hypothesized that the age-related differences are modulated by motivational feedback.

## Materials and Methods

### Participants

A total of 35 young adults (22 females) and 32 older adults (18 females) participated in our study. In the young adult group age ranged from 18 to 37 years with a mean age of 25.3 years (*SD* = 4.3). In the senior adult group age ranged from 60 to 77 years with a mean age of 68.4 years (*SD* = 5.0). Recruitment of subjects was managed by calls for participation at the University of Giessen and in local newspapers. All participants were naive with respect to the purpose of the study and were paid for participating. Any history of ophthalmologic, neurologic, or psychiatric disorders as well as medications presumed to interfere with perceptual capacities were screened out by a detailed interview protocol. In addition, we ran a battery of standard cognitive tasks in order to exclude pathological age-related decline. Visual acuity was measured binocularly confirming normal or corrected-to-normal for all participants. Assessment by the Edinburgh Inventory (Oldfield, 1971) showed right-handedness for the majority of participants. We identified three left-handed young adults and three ambidextrous adults, one in the young adult group and two in the older adult group, respectively. Methods and procedures agreed with the Declaration of Helsinki (World Medical Association, 2013) and were approved by the local ethics committee of the Faculty of Psychology and Sports Science, Justus-Liebig-Universität Gießen. Informed consent was obtained by all participants and protection of data privacy was provided.

### Setup and Stimuli

**FIGURE 1:**
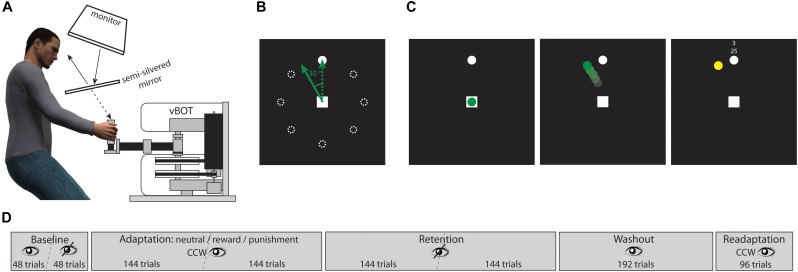
Illustration of setup and stimuli. **(A)** Side view showing the participant in front of the vBOT and looking at the semi-silvered mirror that reflects the image of the monitor. **(B)** Layout of reaching targets; note that only one target was shown at a time, the dotted circles here just illustrate defined positions; the green dotted arrow shows a veridical handle trajectory, the green solid arrow shows the corresponding displayed trajectory in an adaptation trial, i.e., a trajectory rotated counterclockwise by 30°. **(C)** Example adaptation trial, reward condition; at trial start the handle position was indicated by a green dot; the reaching trajectory was displayed online by a moving green dot with a 30° counterclockwise rotation; the reaching endpoint was indicated by a yellow dot and was accompanied by feedback on points based on endpoint error. **(D)** Sequence of experimental blocks, for details see text.

Figure 1A illustrates the setup of our study. We used a two-dimensional planar manipulandum, a vBOT system (Howard et al., 2009), to investigate reaching movements in our participants. Position of the manipulandum handle was recorded at a sampling rate of 1,000 Hz. The vBOT system was integrated in a virtual-reality setup consisting of a monitor/mirror projection system. Stimuli were generated using Matlab with the Psychophysics Toolbox (Brainard, 1997) and displayed on the monitor. The monitor image was projected onto a semi-silvered mirror which produced a virtual image of the display. Participants were seated on a height-adjustable chair so that they were able to take a comfortable position in order to look at the semi-silvered mirror and to grasp the manipulandum handle that was occluded by the mirror. Head position was supported by a chin and a head rest. Distances and angles between monitor, mirror, and the handle plane under the mirror were calibrated such that the virtual image was perceived being at the same plane as the handle.

The layout of possible reaching targets is shown in Figure 1B. At the center of a black background a white square with edge length of 1 cm indicated the starting position of the reaching movement. The reaching target was a white dot with a diameter of 0.3 cm. There were eight possible target positions arranged circularly around the start square at a distance of 8 cm and separated by 45° each.

### Procedure

Before each trial a white square appeared at the center of the display. As soon as the participant encompassed the handle the vBOT system guided it to the square, i.e., the starting position. The square then disappeared and reappeared after 200 ms indicating the trial start. At the same time the reaching target was shown at one of the eight possible positions. In a sequence of eight trials each position occurred once and the order of positions was randomized. All participants used their right hand for steering the handle and were asked to reach to the target as fast and accurately as possible. Response time was defined as the time between the trial start and the handle leaving the starting square, giving the movement onset. Movement time was defined by the time between movement onset and the handle hitting the virtual circle around the starting square on which the target positions were arranged. Visual feedback on the handle position could be provided by a green dot with a diameter of 0.3 cm. An example adaptation trial is shown in Figure 1C.

The complete reaching adaptation procedure consisted of five blocks and is illustrated in Figure 1D. The participants started with a baseline block with 96 trials overall. While in the first half of this block veridical visual feedback on the handle position was provided, no visual feedback was given in the second half. Both halves were separated by a short rest period that was limited to 1 min. In the baseline block participants were supposed to get used to the task under conditions with and without visual feedback. Movement accuracy differences between both feedback conditions were not observed since participants were instructed to reach fast and directly to the targets.

Following the baseline trials, the adaptation block started in which visual feedback on the handle trajectory was provided, but introducing a counterclockwise rotation by 30°. There were a total of 288 adaptation trials split in two halves by a short rest period limited to 1 min. At the end of each trial the final handle position was indicated by a yellow dot with a diameter of 0.3 cm and displayed for 500 ms. Additional motivational feedback was simultaneously displayed next to the reaching target (see Figure 1C). There were three different motivation conditions, i.e., neutral, reward, and punishment. In the neutral condition, two uninformative horizontal lines were displayed. In the reward and the punishment conditions, participants were told in advance that they could earn additional points in some phases of the experiment that would be finally converted to money, i.e., two points yielding one cent. In the reward condition, participants started with zero points and their gain added up across the trials; in the punishment condition participants were given an initial credit of 1,200 points that was reduced by negative points. As motivational feedback both points earned or lost in each trial and totally accumulated points were displayed. Points were based on endpoint error. In the reward condition and the punishment condition, respectively, hitting the target gave 4 points and 0 points, an error < 10° gave 3 points and -1 point, an error < 20° gave 2 points and -2 points, an error < 30° gave 1 point and -3 points, an error > 30° gave 0 points and -4 points. In order to avoid strategic slowing in the adaptation block, a warning signal was shown in trials in which movement time exceeded the 90th percentile of the baseline block. The starting square then turned to red indicating that the reaching movement was too slow. In those trials 0 points and -4 points were given in the reward condition and in the punishment condition, respectively. Assignment of participants to one of the three motivation conditions was done according to the order in which they participated in the study. For every three consecutive young and older adults, respectively, each condition was applied once and was randomly assigned. In the young adult group, there were 13 participants in the neutral condition, 10 participants in the reward condition, and 12 participants in the punishment condition. In the older adult group, there were 10 participants in the neutral condition, 10 participants in the reward condition, and 12 participants in the punishment condition.

The adaptation block was followed by a retention block that comprised 288 trials again split in two halves by a short rest period limited to 1 min. Similar to the second half of the baseline block, here no visual feedback on the handle position was provided. Thus, participants did not receive any performance information that could drive further motor learning processes. This block allowed for investigating retention without feedback on movement errors, i.e., errorless retention. The gradual decay of the adaptive shift in reaching direction specifically characterizes retention.

After retention a washout block with a total of 192 trials was supposed to return the reaching error to the baseline level. Veridical visual feedback of the handle trajectory was given. Finally, relearning was investigated in a readaptation block. There were 96 trials in which the visual feedback was manipulated equivalently to the adaptation block. However, no motivational feedback was provided.

After the last block participants received 8 € per hour plus their additional gain if they were assigned either to the reward or to the punishment condition. Overall the procedure took about 60–90 min and thus the duration was still appropriate for the older participants.

### Data Analysis

Analyses of time measures, i.e., response times and movement times, were based on average data across defined blocks including all trials. Since the washout block was not critical for the investigation of learning parameters, time measures from this block were not considered in detail.

Angular reaching direction was calculated as the difference between the angular target position and the angular handle position at the end of each trial. Thus, in trials without visuomotor rotation reaching directions closer to 0° indicated smaller deviations between the final handle position and the target. In contrast, in adaption trials reaching directions closer to 30° indicated the more accurate movements since feedback on the handle position was rotated counterclockwise by 30°. We excluded trials with extreme reaching directions exceeding 50° or -20°. In addition, trials with response times larger than 1,500 ms were discarded because initiation of the reaching movement was considered as overly delayed putatively due to high-level cognitive processes. Based on these criteria on average 4.4% (*SD* = 2.7%) of all trials had to be discarded. Data from four participants, i.e., one young adult and the three older adults, who showed exclusion rates >10% were excluded from further analyses.

Furthermore, we examined overall time needed for reaching movements in the baseline block in order to identify participants who did not accomplish the task appropriately. We determined within each age group outlier data by inspection of boxplots and considered time measures deviating more than 1.5 times the interquartile range from the range borders as outliers. We identified outlier data for two young adults and two older adults. They were also excluded from further analyses.

Reaching direction data was analyzed using angular measure circular statistics (Berens, 2009). For each participant the average of reaching directions across all trials in the baseline block was removed from the following trials in order to adjust for individual biases in reaching movements. We then derived different parameters that characterize motor learning. As model-free parameters for adaptation and retention we considered the reaching direction shown by the end of the adaptation block and the retention block, respectively. We were here specifically interested in the finally achieved adaptive shift and the persisting retention status. In order to obtain robust measures, we averaged reaching direction across the last third of trials in both the adaptation and the retention block, i.e., across 96 trials each. In addition, we derived model-based parameters using a single-state state-space model to quantify learning and retention parameters (Smith et al., 2006). For the adaptation block, the retention block, and the readaptation block we fitted reaching direction data trial-by-trial based on the following model equations.

y^n=−znt⁢ and⁢ zn+1t=Aznt+B(rn−znt)

y^n gives the angular reaching direction on trial *n*. The current estimated visuomotor rotation associated with the target *t* is given by $z_{n}^{t}\,\;$. *r*n represents the visuomotor rotation on trial *n*. Thus, *r*_n_ - $z_{n}^{t}\,\;$ gives the reaching error on trial *n*. Given the model equation, the parameter *A* provides a measure for retention rate, i.e., persistence of the previous learning state. For the adaptation and readaptation blocks this parameter was not further followed up since in each trial consistent rotated feedback was given excluding substantial forgetting. The parameter *B* provides a measure for adaptation for the experienced reaching error on the previous trial, i.e., the learning rate. Since no visual feedback on the reaching error was provided in the retention block, we set *B* = 0 for fitting. A measure for the learning benefit based on repeated exposure to the visuomotor rotation was given by the difference between the learning rates in the readaptation block and the adaptation block, i.e., savings.

By evaluating boxplots for the different learning parameters and applying the same criteria as described above we determined further outlier data for each age group. Extreme parameters suggested that the corresponding participants showed insufficient compliance with the task instructions, applied specific strategies for accomplishing the task, or were affected early by fatigue. In the adaptation block, two young participants showed an extremely high or low learning performance, respectively. For another young participant we determined almost complete persistence of adaptation in the retention block. Finally, an older adult showed extremely low savings, i.e., benefit from repeated learning, in the readaptation block suggesting pronounced fatigue across the experiment. In order to reduce noise in our data we excluded the identified participants from further analyses.

The exploratory data analysis and the corresponding exclusion of participants reduced our dataset to 29 young adults and 26 older adults. We tested whether exclusion of participants was related to age group or motivational condition. There was no significant bias in exclusion that could complicate analyses of the final dataset, Φ = 0.63, *p* = 0.091.

If not stated otherwise, data were analyzed using two-way factorial ANOVAs with the between-subjects factor *age group* (young adults vs. older adults) and the between-subjects factor *motivation condition* (neutral vs. reward vs. punishment). Significant main effects were followed up by planned simple contrasts comparing both the reward and the punishment condition to the neutral condition, respectively. Significant interaction effects were scrutinized by one-way ANOVAs for each age group with the factor *motivation condition*. *Post hoc* contrasts were Bonferroni–Holm corrected for multiple comparisons. A significance level of α = 0.05 was used for all statistical analyses. Descriptive values are given as means ± SEMs.

## Results

We aimed to investigate how motivational incentives modulate age-related differences in motor learning. Performance of a young adult group and an older adult group was assessed in a reaching task that required fast shooting movements toward visual targets.

**FIGURE 2:**
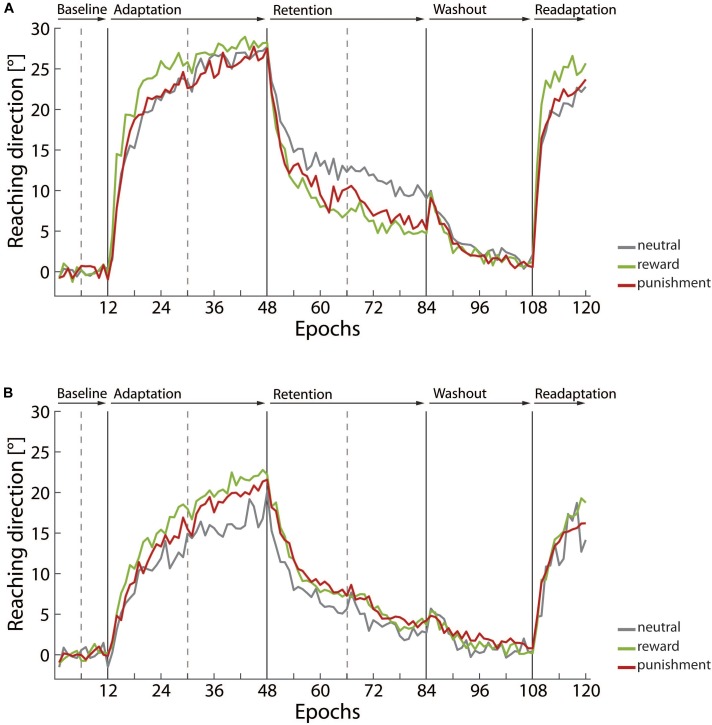
Averaged reaching direction across the experimental blocks for **(A)** the young adult group and **(B)** the older adult group, respectively. Epoch data, i.e., average across eight trials, is used for plotting reaching direction. Data is given in gray for neutral condition groups, in green for the reward condition groups, and in red for the punishment condition groups.

Adaptive processes were triggered by manipulation of visual feedback on the individual reaching trajectories. The applied visuomotor rotation paradigm robustly elicited adaptation of reaching direction in both age groups. Figure 2 shows the averaged angular reaching direction across the experimental blocks for young adults (Figure 2A) and older adults (Figure 2B). Reaching direction is plotted epochs that give the average across eight reaching movements.

The illustration shows an overall similar pattern in both age groups. In the baseline block, reaching direction fluctuated around 0°, i.e., a perfect match between final handle position and the target. Considering the complete sample, we indeed determined a counterclockwise bias, -0.91 ± 0.18°, that significantly deviated from 0°, *t*(54) = -5.11, *p* < 0.001, *d* = -1.39. This bias was most likely due to our procedure requiring that the right hand was used for the reaching movements. A one-way ANOVA with the factor *motivation condition* yielded no evidence that this bias was affected by the motivation condition participants were assigned to, *F*(2,52) = 0.47, *p* = 0.631, η^2^ = 0.02. As described above in the Materials and Methods section we adjusted reaching direction data for the bias by removing the average across the baseline block from the following trials.

In the adaptation block, in which visual feedback on reaching trajectories was systematically rotated counterclockwise by 30°, reaching direction shifted across the epochs so that the angular error between the final handle position and the target was reduced. In the retention block without visual feedback the adaptive shift in reaching direction declined, but was not completely abolished. The washout block with veridical visual feedback on reaching trajectories finally brought the reaching direction back close to 0°. Picking up rotated visual feedback in the readaptation block again elicited adaptive shifts of reaching direction. In summary, young and older adults showed the expected pattern of shifts in reaching direction that were supposed to be triggered by the experimental procedure. However, the comparison between Figure 2A, i.e., young adults, and Figure 2B, i.e., older adults, points to substantial differences in magnitude of shifts observed in both age groups and in particular to specific effects of motivational feedback provided in the adaptation. In the following, detailed results for motor learning parameters in the adaptation block, the retention block, and the readaptation block are presented.

### Visuomotor Learning

In the adaptation block, participants who were assigned to either the reward or the punishment condition had the opportunity to earn points based on endpoint error of their reaching movements (see “Materials and Methods” section). Thus, better motor learning yielded more points in the reward condition and reduced lost points in the punishment condition, respectively. For both conditions we determined significant age effects on the point outcome. In the reward condition, young adults won on average 2.72 ± 0.04 points per trial, but older adults won only 1.90 ± 0.12 points per trial [*t*(11.1) = 6.33, *p* < 0.001, *d* = 2.79]. In the punishment condition, young adults lost on average -1.55 ± 0.07 points per trial whereas older adults lost -2.30 ± 0.12 points per trial [*t*(18) = 5.46, *p* < 0.001, *d* = 2.57]. These differences indicated an age-related disadvantage in motor learning that was corroborated by analysis of the specified learning parameters in the adaptation block.

**FIGURE 3:**
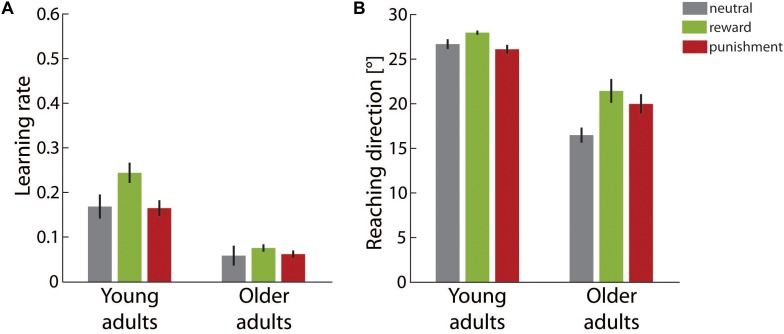
Learning results in the adaptation block. **(A)** Average learning rates in the different motivation condition groups for young and older adults. **(B)** Average final reaching direction in the different motivation condition groups for young and older adults. Error bars depict ±1 SEM.

Figure 3A illustrates the learning rate for both adult groups in the different motivational feedback conditions. We determined significant main effects of *age group*, *F*(1,49) = 66.19, *p* < 0.001, η^2^ = 0.56, and *motivation condition*, *F*(2,49) = 3.84, *p* = 0.028, η^2^ = 0.14. The interaction effect of both factors did not reach significance, *F*(2,49) = 1.74, *p* = 0.185, η^2^ = 0.07. Older adults showed lower learning rates than young adults in all motivational feedback conditions.

The main effect of *motivation condition* was followed up by two planned contrasts comparing the neutral condition to the reward and the punishment condition, respectively. Whereas the learning rate was significantly higher in the reward condition than in the neutral condition, contrast estimate 0.05 ± 0.02, *p* = 0.046, punishment did not affect the learning rate, contrast estimate <0.01 ± 0.02, *p* = 0.991.

The final adaptive state at the end of the adaptation block is shown in Figure 3B. Reaching direction in the last third of trials was significantly affected by *age group*, *F*(1,49) = 115.63, *p* < 0.001, η^2^ = 0.70, and *motivation condition*, *F*(2,49) = 6.05, *p* = 0.005, η^2^ = 0.20. However, the main effects were qualified by a significant interaction of both factors, *F*(2,49) = 3.27, *p* = 0.046, η^2^ = 0.12. As *post hoc* analyses we ran separate one-way ANOVAs with the factor *motivation condition* for both age groups. For young adults, the effect *motivation condition* just failed to reach significance, *F*(2,26) = 3.18, *p* = 0.058, η^2^ = 0.20. In contrast, in the older adult group significant differences between the motivational feedback groups were supported, *F*(2,23) = 4.10, *p* = 0.030, η^2^ = 0.26. Planned contrasts showed a more pronounced adaptive shift in the reward condition than in the neutral condition, contrast estimate 4.95 ± 1.74°, *p* = 0.018. The comparison between the punishment condition and the neutral condition neared significance, contrast estimate 3.50 ± 1.74°, *p* = 0.056.

In summary, our findings in the adaptation block consistently corroborated expected reduced motor learning capacities in older adults when compared with young adults. In addition, analyses yielded evidence that motivational feedback modulates motor adaptation in both age groups. Results from model-based and model-free analyses did not overlap completely, but overall supported a similar pattern. Faster learning was induced by rewarding feedback. Punishing feedback was less efficient and did not boost learning significantly relative to neutral feedback. This pattern was observed for young adults as well as for older adults. However, at the end of the adaptation block it only neared significance for younger adults. Since the adaptive shift in reaching direction then was close to complete for young adults, the attenuation of the motivational effect might be due to a ceiling effect.

### Visuomotor Retention

**FIGURE 4:**
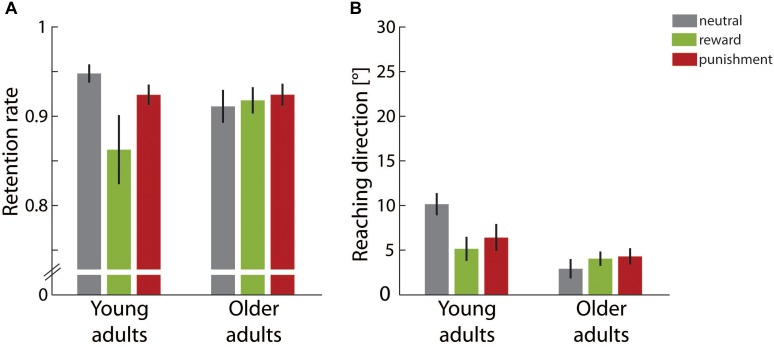
Retention results. **(A)** Average retention rates in the different motivation condition groups for young and older adults. **(B)** Average final reaching direction in the different motivation condition groups for young and older adults. Error bars depict ±1 SEM.

In the retention block no visual feedback was provided and thus it allowed for investigating errorless retention. Figure 4A shows the retention rate for both adult groups in the different motivational feedback conditions. The lower the retention rate was, the faster the reaching direction shift built up across the adaptation block decayed in the absence of performance feedback. The two-factorial ANOVA yielded a significant main effect for *motivation condition*, *F*(2,49) = 3.20, *p* = 0.049, η^2^ = 0.12, but not for *age group*, *F*(1,49) = 0.21, *p* = 0.650, η^2^ < 0.01. In addition, a significant interaction effect of both factors was found, *F*(2,49) = 3.70, *p* = 0.032, η^2^ = 0.13. *Post hoc* one-way ANOVAs for each age group showed that retention rate significantly varied across motivational feedback conditions in young adults, *F*(2,26) = 5.47, *p* = 0.010, η^2^ = 0.30, but not in older adults, *F*(2,23) = 0.20, *p* = 0.825, η^2^ = 0.02. For the young adults, we further clarified that the retention rate was significantly lower in the reward condition than in the neutral condition, contrast estimate -0.09 ± 0.03, *p* = 0.006. However, retention rates in the punishment condition and in the neutral condition were equivalent, contrast estimate -0.02 ± 0.02, *p* = 0.317.

Figure 4B gives the final reaching direction in the last third of the retention block. For this parameter we determined a significant main effect of *age group*, *F*(1,49) = 13.02, *p* = 0.001, η^2^ = 0.21, but not for *motivation condition*, *F*(2,49) = 1.28, *p* = 0.287, η^2^ = 0.05. Again a significant interaction effect of both factors, *F*(2,49) = 3.66, *p* = 0.033, η^2^ = 0.13, qualified main effect results. *Post hoc* one-way ANOVAs again yielded a significant effect of motivation condition for young adults, *F*(2,26) = 3.95, *p* = 0.032, η^2^ = 0.23, but not for older adults, *F*(2,23) = 0.57, *p* = 0.572, η^2^ = 0.05. Young adults showed a significantly lower retention rate in both the reward condition, -5.01 ± 1.96°, *p* = 0.034, and in the punishment condition, -0.02 ± 0.02°, *p* = 0.044, when compared to the neutral condition.

Overall results in the retention block pointed to an interaction effect between motivational feedback and age group. For young adults motivational feedback during initial learning was detrimental for retention performance. In particular, rewarding feedback induced significantly faster forgetting and was associated with a smaller persisting adaptive shift at the end of the retention block. Punishing feedback, though not triggering performance differences during initial learning, similarly was associated with a smaller persisting adaptive shift. In contrast to these finding for younger adults, older adults’ retention performance was not affected by the motivational condition during initial learning.

### Visuomotor Relearning

We investigated relearning rates in the readaptation block in order to clarify how motivational effects on adaptation and retention affect relearning in the different age groups. By the end of the washout block reaching direction was close to 0° in all experimental groups (compare Figure 2). Using the last 16 trials of the washout block as reference, we however, found a persisting shift in reaching direction for the complete sample, 0.80 ± 0.23°, *t*(54) = 3.56, *p* = 0.001, *d* = 0.97. This means that no complete washout was accomplished by the 192 trials with veridical visual feedback. The persisting shift, though, did not correlate with learning rate in the following readaptation block, *r*(55) = 0.05, *p* = 0.729. Furthermore, factorial analysis yielded no evidence for significant main effects of *age group, F*(1,49) = 2.10, *p* = 0.153, η^2^ = 0.04, or *motivation condition*, *F*(2,49) = 0.11, *p* = 0.897, η^2^ = 0.01, on the reaching direction by the end of the washout block. In addition, the interaction effect was also not significant, *F*(2,49) = 0.82, *p* = 0.446, η^2^ = 0.03. Thus we suggest that any differences in relearning are rather unlikely to be induced by incomplete washout, but can be linked to differences in previous adaptation and retention.

**FIGURE 5:**
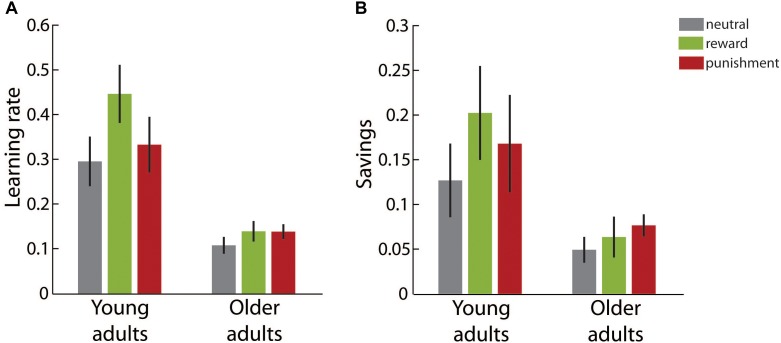
Learning in the readaptation block. **(A)** Average learning rates in the different motivation condition groups for young and older adults. **(B)** Average savings, i.e., the difference between the learning rates in the readaptation block and the adaptation block, in the different motivation condition groups for young and older adults. Error bars depict ±1 SEM.

Figure 5A illustrates the learning rate in the readaptation block for each age group in the different motivational feedback conditions. Please note that during readaptation no additional motivational feedback was provided. We determined a significant main effect of *age group*, *F*(1,49) = 36.71, *p* < 0.001, η^2^ = 0.43. Older adults showed lower learning rates during readaptation than young adults in all motivational feedback conditions. Neither the main effect of *motivation condition*, *F*(2,49) = 1.86, *p* = 0.167, η^2^ = 0.07, nor the interaction effect, *F*(2,49) = 1.02, *p* = 0.369, η^2^ = 0.04, reached significance.

In Figure 5B savings, i.e., the difference between the learning rate in the readaptation block and the learning rate in the adaptation block, are shown. Savings represent the learning benefit when participants adapt to a visuomotor manipulation that they have already experienced before. In all age groups and motivational feedback condition, respectively, savings were significantly larger than zero (all *p*s ≤ 0.017). We found a significant main effect of age group, *F*(1,49) = 11.06, *p* = 0.002, η^2^ = 0.18, but neither a significant main effect of *motivation condition*, *F*(2,49) = 0.73, *p* = 0.489, η^2^ = 0.03, nor a significant interaction effect, *F*(2,49) = 0.35, *p* = 0.710, η^2^ = 0.01. Older adults benefitted less from the previous learning experience than young adults.

In summary, congruent with our findings in the adaptation block learning rate in the readaptation block was significantly lower in older than in young adults. In addition, older adults showed smaller benefits from repeated learning indicated by savings. However, there was no significant evidence that motivational feedback during initial learning affected learning performance during readaptation. Although visual inspection of Figure 5 suggests an overall beneficial effect of motivational feedback on learning rate and savings across both age groups, differences did not reach significance given pronounced variability of parameters in this last experimental block.

### Response and Movement Times

Finally, we analyzed response and movement times in order to clarify whether the effects of motivation condition during reaching adaptation are linked to differential movement strategies. Table 1 summarizes the data for young and older adults.

**Table 1:** Response times (RT) and movement times (MT) in the different motivation condition groups for young and older adults; data is given for the baseline block and the main experimental blocks.

	Younger adults		Older adults	
		
	RT (ms)	MT (ms)	RT (ms)	MT (ms)
**Baseline**				
Neutral	403 ± 10	123 ± 6	481 ± 11	144 ± 8
Reward	413 ± 19	131 ± 14	514 ± 18	137 ± 10
Punishment	424 ± 10	144 ± 13	529 ± 38	140 ± 11
**Adaptation**				
Neutral	441 ± 20	121 ± 9	542 ± 10	142 ± 16
Reward	415 ± 34	120 ± 13	523 ± 22	143 ± 9
Punishment	439 ± 19	137 ± 13	567 ± 41	138 ± 7
**Retention**				
Neutral	408 ± 16	109 ± 5	484 ± 19	125 ± 7
Reward	396 ± 22	117 ± 12	519 ± 36	134 ± 8
Punishment	432 ± 22	116 ± 9	523 ± 31	123 ± 6
**Readaptation**				
Neutral	421 ± 18	117 ± 6	557 ± 34	142 ± 18
Reward	419 ± 30	130 ± 16	554 ± 66	140 ± 10
Punishment	431 ± 22	137 ± 12	550 ± 35	150 ± 16


Time measures were specifically of interest in the adaptation block, the retention block, and the readaptation block since the learning parameters were derived from these blocks. Data from the baseline block was used for checking systematic differences between our participant groups that were not due to the specific motivational manipulations. For each block we ran 2 (*age group*) × 3 (*motivation condition*) ANOVAs on both response times and movement times, respectively.

In the baseline block we only determined a significant main effect of *age group* on response times, *F*(1,49) = 28.1, *p* < 0.001, $\eta_{p}^{2}$ = 0.36. Older adults started their movements significantly slower than young adults. Most importantly, response times were affected neither by a main effect of *motivation condition* nor an interaction effect between both factors (*p*s ≥ 0.292). Analysis of movement times yielded no significant effects. Thus, we found no evidence for systematic biases in the baseline block that could limit interpretation of our results in the later blocks.

ANOVAs for the main experimental blocks consistently showed significant main effects of *age group* on response times [adaptation block: *F*(1,49) = 24.0, *p* < 0.001, $\eta_{p}^{2}$ = 0.33; retention block: *F*(1,49) = 19.2, *p* < 0.001, $\eta_{p}^{2}$ = 0.28; readaptation block: *F*(1,49) = 16.2, *p* < 0.001, $\eta_{p}^{2}$ = 0.25]. However, reaction times were not affected by main or interaction effects of *motivation condition* (all *p*s ≥ 0.463). Analysis of movement times again yielded no significant effects across all considered blocks. In summary, these results indicated that motivational effects on the different learning parameters could not be explained by specific strategies for movement initiation or execution that would have affected time measures. Congruently, in neither age group we found significant correlations between the time measures and the relevant learning parameters in the specific experimental blocks (all *p*s ≥ 0.10).

## Discussion

This study was concerned with motivational modulation of motor learning. We investigated reaching adaptation in a well-established visuomotor rotation paradigm that is known to robustly induce adaptive movement shifts (e.g., Krakauer et al., 2000; Krakauer, 2009). We coupled movement endpoint error experienced during adaptation to neutral, rewarding, or punishing feedback. Comparing reaching performance in a group of young adults and a group of healthy older adults, we explored whether motivational incentives modulate age-related decline in motor learning.

Visuomotor perturbations in our paradigm triggered consistent recalibration of reaching direction across all participants. However, motor plasticity was found significantly reduced in older adults. This result corroborated findings from previous studies using similar reaching adaptation procedures (e.g., Buch et al., 2003; Bock, 2005; Heuer and Hegele, 2008b, 2009). It has been suggested that in particular an age-related vulnerability of explicit, strategic components contributing to adaptation drive these age effects; in contrast implicit components have found to be preserved across the lifespan and putatively stabilize motor learning (Heuer and Hegele, 2009; Heuer et al., 2011; Huang et al., 2017). Although both implicit and explicit components were supposed to be involved in the observed recalibration of reaching direction (see Taylor et al., 2014), our procedure putatively favored the flexible application of explicit strategies. The visuomotor perturbation was introduced abruptly and no supporting instructions were given. In addition, we used varying target directions for which explicit, but not implicit components generalize (Heuer and Hegele, 2011). Given the particular vulnerability of explicit adaptation components, we suggest that the age-related attenuation of reaching adaption primarily reflects reduced availability or use of explicit strategies.

Although, we determined an overall detrimental age effect on reaching adaptation, motivational modulation of motor learning was found to be stable across both age groups. In particular error-based reward boosted learning during acquisition, while punishment was less efficient. Young and older adults equivalently showed faster learning rates with rewarding feedback than with neutral feedback. The final adaptive state as measured by reaching direction at the end of the adaptation block varied between the feedback conditions in older adults, but not in young adults. Older adults showed larger adaptive shifts with rewarding feedback. Also punishing feedback tended to have a positive effect, though significance was failed. We speculate that in young adults motivational modulation was obscured at the end of the adaptation block because their adaptive shifts in reaching direction then were close to complete, resulting in a ceiling phenomenon. Indeed, our paradigm seemed to trigger especially high learning rates in young adults when compared with previous studies using similar visuomotor rotations by 30°. In the neutral condition we determined an average learning rate of 0.16 for young adults, whereas e.g., in the study of Galea et al. (2015) a learning rate of about 0.06 was described. Thus, by the end of the adaptation block the additional beneficial impact of motivational feedback was probably limited. In summary, our findings supported a significant boost of motor learning induced by rewarding feedback. This motivational modulation is preserved across the adult life span and qualifies as a potentially compensating mechanism for age-related functional decline.

Beneficial effects of reward on motor learning are congruent with recently accumulating evidence showing increased learning rates when rewarding feedback is provided (Nikooyan and Ahmed, 2015; Kojima and Soetedjo, 2017). Even in participant groups with presumably reduced processing resources, i.e., stroke patients, reward was found to enhance adaptive processes (Quattrocchi et al., 2017). However, our findings on punishment effects on reaching adaptation deviate from previous reports. Galea et al. (2015) as well as Quattrocchi et al. (2017) determined significant beneficial effects of punishing feedback during adaptation in a reaching task. The absence of an equivalent effect in our study might be explained by procedural details. It has been speculated that the observed punishment effects were driven by loss avoidance. However, actual loss avoidance is strongly shaped by contextual parameters (Palminteri et al., 2015; Sternad and Körding, 2015). In our procedure, participants assigned to the punishment condition started with an initial credit of 1,200 points which was reduced by losses and converted to money only by the end of the experiment. Either the magnitude of the initial credit or the rather abstract concept of points might have buffered loss avoidance. In the previous studies, initial credit was immediately provided in concrete units of money, i.e., 12 and 50 $, respectively. Thus, we tentatively assume that the operationalization of punishment feedback constrained its functional efficiency.

In our experimental procedure, the adaptation block was followed by a block in which participants did not receive any feedback on their reaching trajectories. Thus, we were able to explore errorless retention in both adult age groups and in particular in the different motivational feedback conditions. Our data showed an intriguing interaction effect between age group and motivational feedback. Corroborating previous results we found no main effect of age group on retention rates (e.g., Bock, 2005; Heuer and Hegele, 2008a,b; Hegele and Heuer, 2013). However, while motivational feedback during learning was detrimental to retention in young adults, older adults’ retention performance did not vary across motivational conditions.

The results for the young adults provided further support that beneficial effects of motivational feedback during adaptation do not necessarily transfer to retention (compare Galea et al., 2015; Steel et al., 2016). In addition, a detrimental effect on performance might be triggered by withdrawal of extrinsic incentives so that intrinsic motivation is reduced (for review, see Deci et al., 1999). Galea et al. (2015) indeed found that a beneficial effect of punishment during adaptation disappeared during following retention, whereas reward only became efficient for retention. They interpreted this pattern as evidence for independent mechanisms driving learning and retention in reaching adaptation. We did not replicate the positive effect of reward on retention; in contrast our results showed that in particular reward was detrimental to retention rates. However, since efficiency of reward and punishment we found during adaptation substantially deviated from the previous study, we refrain from direct comparison and suggest that a more elaborated clarification is needed.

Discrepant effects of motivational feedback for both age groups might indicate that motivational feedback modulates differential adaptation components in young and older adults. Körding et al. (2007) proposed that sensorimotor adaptation can be modeled as a combination of fast and slow processes. Fast processes are supposed to drive rapid adaptive changes which are prone to rapid decay when visuomotor feedback is withdrawn. Slow processes, in contrast, contribute to adaptation and decay only slowly (Ethier et al., 2008). We suggest that the distinction between explicit and implicit components involved in adaptation can be linked to the distinction between fast and slow processes, respectively (compare Huang et al., 2017). It can be supposed that adaptation primarily driven by explicit strategies decays faster, whereas contributions of implicit components result in movement shifts more robust to decay. Given this pattern we speculate that motivational feedback in particular boosts explicit adaptation components in young adults, resulting in more pronounced decay. In contrast, in older adults the availability and use of explicit strategies during reaching adaptation have been shown to be reduced (Hegele and Heuer, 2010c; Heuer et al., 2011). Thus, in this age group motivational feedback might primarily act on implicit adaptation components that decay slowly and stabilize retention. Since we did not assess the differential contributions of explicit and implicit components directly in our study, this link has to remain speculative, but points to a highly relevant dissociation that could underlie observed age effects.

Readaptation to the previously experienced visuomotor rotation was subject to significant age effects, but modulation by motivational feedback failed to reach significance. Older adults showed again lower learning rates and benefitted less from repeated learning as indicated by savings. Savings represent the ability of initial learning to enhance later relearning (Smith et al., 2006). Although both implicit and explicit processes are involved in readaptation, explicit processes, e.g., recognition of the previously experienced rotation, can be assumed to play a dominant role. Thus, our results for readaptation appear consistent with the age-related vulnerability of explicit, strategic mechanisms contributing to adaptation. Descriptive inspection of our data supported persistence of the beneficial effects of reward on learning in both age groups. Also punishment absolutely enhanced learning rates and savings during readaptation. Even though these observed effects were not statistically significant and therefore elude authoritative conclusions, overall the beneficial trend of motivational feedback agrees with previous findings (Galea et al., 2015; Quattrocchi et al., 2017).

There was no evidence that within each age group motivational feedback affected response or movement times. It has been recently suggested that the use of explicit strategies during adaptation is associated with increasing latencies (Benson et al., 2011; Fernandez-Ruiz et al., 2011; Sülzenbrück and Heuer, 2012). Thus, varying latencies across the different feedback conditions could indicate differential contributions of implicit and explicit adaptation components. Since slowing represents a core primitive of functional changes with increasing age (Salthouse, 1996), a comparison of latency differences between age groups did not allow for specific conclusions. Most importantly, neither in the young nor in the older adult group motivational effects on learning parameters were linked to motivational feedback. We propose that motivational feedback does not substantially shift the balance between explicit and implicit components contributing to adaptation, but affects the components that are particularly efficient in the specific age group, i.e., explicit components in young adults and implicit components in older adults.

Several neuronal substrates have been suggested to functionally contribute to motor learning. Candidate structures in particular include the cerebellum and the motor cortex (see e.g., Li et al., 2001; Krakauer et al., 2004). There is evidence that the cerebellum contributes to error-based learning, whereas cortical areas are crucial for retention of motor adaptation (Hadipour-Niktarash et al., 2007; Galea et al., 2011; Orban de Xivry et al., 2011). Aging is supposed to negatively affect both processes since cortical as well as cerebellar structures are subject to pronounced age-related decline (Jernigan et al., 2001; Sowell et al., 2003; Raz et al., 2005). However, differential contributions to reduced motor learning capacities during aging appear far from conclusive. For example, impaired use of explicit learning strategies has been identified as characteristic of age effects on motor learning. At the same time, this capacity has been found preserved in cerebellar patients (Taylor et al., 2010). Consistent with previous findings, our results indicate detrimental age effects on learning as well as retention during reaching adaptation, but specific underlying neuronal substrates remain ambiguous. Motivational modulation of motor learning is critically conveyed by dopamine (Montague et al., 2004; Hosp and Luft, 2013). Although dopaminergic transmission is known to decline with increasing age (for review, see Bäckman et al., 2010), our results provided evidence for preserved motivational modulation of reaching adaptation. They further emphasized that robust behavioral resources can be available despite significant physiological changes during healthy aging. Age-related pathological processes, though, might challenge these resources.

To conclude, our study provides evidence that motivational modulation of reaching adaptation is preserved during healthy aging. Although older adults showed reduced motor learning capacities and typical slowing effects, they substantially benefitted from motivational incentives during learning. Most importantly, this benefit applied to learning as well as retention of adaptive shifts. We suggest that motivational feedback can be used as a potentially compensatory mechanism during functional aging. Although our data corroborate a persistent age-related decline in visuomotor plasticity, motivational feedback attenuates performance differences. Our findings further emphasize the complexity of processes that contribute to motor adaptation (compare also Krakauer and Mazzoni, 2011). There is a need to disentangle these processes in order to evaluate their behavioral significance, in particular in populations that face functional challenges.

## Author Contributions

JH designed the experiments, performed the research, analyzed the data, and wrote the manuscript. MH designed the experiments and wrote the paper. JB designed the experiments, analyzed the data, and wrote the manuscript.

## Conflict of Interest Statement

The authors declare that the research was conducted in the absence of any commercial or financial relationships that could be construed as a potential conflict of interest.
